# Supplementary material for: A set of multi-entry identification keys to African frugivorous flies (Diptera, Tephritidae)
Source: Zookeys. 2014 Jul 24;(428):97–108. doi: 10.3897/zookeys.428.7366 (PMC4143993; doi:10.3897/zookeys.428.7366)
Supplement: Supplementary material 6 — Key to Ceratitis [file zookeys-428-097-s006.zip › SF6_ZooKeys_key to Ceratitis/key/SF6_ZooKeys_key to Ceratitis/Media/Html/Ceratitis podocarpi.htm]

Ceratitis (Pterandrus) podocarpi (Bezzi)


***Ceratitis*** **(*Pterandrus*) *podocarpi*
(Bezzi)**

*Pterandrus* *podocarpi*
Bezzi, 1924a: 476.

Body
length: 4.33 (3.55-4.75) mm; wing length: 4.39 (3.70-4.70) mm.

Male

Head: Antenna yellowish orange. First flagellomere three
times as long as pedicel. Arista with very short rays; ventral rays absent or
shorter and sparser than dorsal rays basally. Frons on ventral half swollen;
yellow, lateral margin pale yellow; with short scattered setulae largely of
same color as frons. Frontal setae slightly less developed than other setae.
Face yellowish white; gena darker yellow. Genal seta and setulae dark, well
developed.

Thorax: Postpronotal
lobe white, with brown spot. Scutal pattern, ground color orange-brown,
microtrichose areas with silvery or ashgray shine; spots brown, prescutellar
yellow markings separate. Scapular setae dark. Scutellum yellow, basally with
two separate dark spots, apically with three separate black spots, extending to
basal 0.33, touching basal spots or almost so. Anepisternum along ventral
margin only darker yellow; setulae pale, except few dark setulae ventrally.

Legs:
Yellowish orange except where otherwise noted; setation typical for subgenus,
mixed pale and dark. Foreleg: femur posteriorly with weakly developed and
dispersed bush of long dark setulae along entire length, posterodorsal setulae
longer; ventral setae dark. Midleg: femur anteriorly with row of short dark
setae on basal half, not always conspicuous; ventrally with few dispersed long
pale setulae, distally darker setae; tibia darker and dilated in distal 0.66;
ventrally and dorsally with black feathering along distal half, anterodorsally
extra row of black feathering along distal half to 0.66, except apical 0.17.
Hindleg: femur at apical 0.25 with longer setulae dorsally and ventrally.

Wing:
bands yellowish brown. Marginal and discal bands with interruption, or partly
joined; cubital band free; medial band absent (sometimes weakly present as free
band); crossvein R-M just beyond middle of discal cell. Apex of vein R_1_
distal to level of crossvein R-M. Crossvein DM-Cu oblique anterobasally.

Abdomen:
Mostly yellow. Tergite 1 with brown patches across posterior margin. Tergites 2
and 4 with silvery transverse band along� posterior half, brownish colored
anteriorly as two large spots, sometimes wholly brownish colored. Tergite 3
largely yellowish brown. Tergite 5 yellow, with brown patches along anterior
and posterior margin. Setation typical for subgenus. Male epandrium in lateral
view with lateral surstylus slender and elongate, anterior lobe strongly
pronounced.

Female

As
male except for the following characters: Frons yellow with few dark patches.
Frontal setae well developed. Anepisternum along ventral margin dark brown,
sometimes extended to ventral 0.33. Crossvein DM-Cu oblique posterobasally.
Legs without feathering, pilosity dark; femora orange-brown; forefemur
posteroventrally with dark pilosity. Abdominal tergite 5 with silvery
transverse band along� posterior margin; sometimes abdomen completely brown
with silvery bands. Oviscape shorter than preabdomen. Aculeus about six times
longer than wide; tip pointed and lateral margin concave.

(Description
after De Meyer & Freidberg, 2006)
